# Mitochondrial Bioenergetics, Redox Balance, and Calcium Homeostasis Dysfunction with Defective Ultrastructure and Quality Control in the Hippocampus of Aged Female C57BL/6J Mice

**DOI:** 10.3390/ijms24065476

**Published:** 2023-03-13

**Authors:** Angie K. Torres, Claudia Jara, Jesús Llanquinao, Matías Lira, Daniela Cortés-Díaz, Cheril Tapia-Rojas

**Affiliations:** 1 Laboratory of Neurobiology of Aging, Centro de Biología Celular y Biomedicina (CEBICEM), Universidad San Sebastián, Santiago 7510156, Chile; 2 Centro Científico y Tecnológico de Excelencia Ciencia & Vida, Avda. Zañartu 1482, Ñuñoa, Santiago 7780272, Chile

**Keywords:** mitochondria, aging, hippocampus, bioenergetic, mitochondrial function

## Abstract

Aging is a physiological process that generates progressive decline in many cellular functions. There are many theories of aging, and one of great importance in recent years is the mitochondrial theory of aging, in which mitochondrial dysfunction that occurs at advanced age could be responsible for the aged phenotype. In this context, there is diverse information about mitochondrial dysfunction in aging, in different models and different organs. Specifically, in the brain, different studies have shown mitochondrial dysfunction mainly in the cortex; however, until now, no study has shown all the defects in hippocampal mitochondria in aged female C57BL/6J mice. We performed a complete analysis of mitochondrial function in 3-month-old and 20-month-old (mo) female C57BL/6J mice, specifically in the hippocampus of these animals. We observed an impairment in bioenergetic function, indicated by a decrease in mitochondrial membrane potential, O_2_ consumption, and mitochondrial ATP production. Additionally, there was an increase in ROS production in the aged hippocampus, leading to the activation of antioxidant signaling, specifically the Nrf2 pathway. It was also observed that aged animals had deregulation of calcium homeostasis, with more sensitive mitochondria to calcium overload and deregulation of proteins related to mitochondrial dynamics and quality control processes. Finally, we observed a decrease in mitochondrial biogenesis with a decrease in mitochondrial mass and deregulation of mitophagy. These results show that during the aging process, damaged mitochondria accumulate, which could contribute to or be responsible for the aging phenotype and age-related disabilities.

## 1. Introduction

Aging is a natural process characterized by physiological dysfunction in response to accumulative damage. Physiological impairment is a consequence of the deterioration of diverse organs and tissues, including the brain [1]. In the aged brain, a loss of connectivity and less robust synapses are observed, causing deterioration of motor and cognitive function, depending on the brain region compromised [2]. Specifically, in the hippocampus, the brain region controlling the spatial and recognition learning and memory processes, aging gradually reduces these abilities because of cellular deficiencies and reduced synaptic communication [3].

One of the most studied age-related cell defects found in the literature is mitochondrial dysfunction. Mitochondrial dysfunction is a hallmark of aging [4]. Mitochondria are dynamic organelles that respond to physiological stimuli. Their main functions include ATP production, Ca^2+^ homeostasis, and redox balance [5], all essential for proper cell functioning. Mitochondria produce ATP through oxidative phosphorylation (OXPHOS), reactive oxygen species (ROS) as a secondary product of complexes I and III [6], and the mitochondrial membrane potential (MMP) of complex I, III, and IV [7] necessary for the correct functioning of mitochondria. Dysfunction of the electron transport chain (ETC) decreases MMP and produces high ROS levels, causing oxidative damage and bioenergetic deficits. Excessive ROS is counteracted by different antioxidant pathways, such as Nrf2 signaling [8]. Mitochondria also regulate calcium homeostasis, uptaking Ca^2+^ in response to increased cytosolic Ca^2+^ concentration [9], mainly via the mitochondrial calcium uniporter (MCU) [10,11]. However, increased mitochondrial Ca^2+^ uptake opens the mitochondrial permeability transition pore (mPTP) [9], leading to mitochondrial swelling and apoptosis [12]. Cyclophilin D (Cyp-D) is fundamental for pore opening [13], and other important components are oligomycin sensitivity-conferring protein (OSCP), a subunit of ATP synthase [14], adenine nucleotide translocator (ANT), and voltage-dependent anion channel (VDAC). Thus, each mitochondrial function is highly controlled in order to maintain cell functionality. Mitochondrial function is also regulated by different processes, including fusion and fission events [15,16,17]. Fusion is mediated by mitofusin 1 (Mfn1), mitofusin 2 (Mfn2), and Opa1 proteins, whereas fission is controlled by Drp1 and Fis1, among others [18]. In addition, the mitochondrial state is regulated by degrading damaged mitochondria and the generation of new mitochondria. Mitophagy is a mitochondrial quality control process that mediates the degradation of depolarized mitochondria through autophagy [19]. Mitochondrial biogenesis is mediated by the co-activator PGC-1α through the stability of the mtDNA [20,21,22]. Altogether, these processes work in conjunction to preserve the mitochondrial and cellular integrity.

With regard to aging, diverse studies have reported that mitochondria lose their functions and that their morphology is aberrant in both the central and peripherical nervous systems in mice [23,24,25,26]. However, this loss of function in the hippocampus in the same murine model has not been investigated in depth. Indeed, in the literature, diverse studies demonstrate some alteration in mitochondrial structure [27] and function in the hippocampus [28], but they were carried out on different models, such as rat and mouse, or using different strains of mice, sex, and ages [29,30,31]. Therefore, the lack of a complete study showing all the alterations that occur in mitochondria during aging in the same animal model, strain, sex, and age has hindered the understanding of the severity of mitochondrial dysfunction in the hippocampus.

Aging research is possible with in vivo study using experimental models, from invertebrate to vertebrate models, which have varied lifespans [32]. Within vertebrates, C57BL/6 mice are the most commonly used and characterized strain for the study of aging [33]. This strain has an average lifespan of approximately 24 months. From 18 to 24 months old, they are considered aged, as this age range is equivalent to 56 to 69 years in human years [34]. In this research, we analyzed mitochondrial dysfunction during aging in female 20-month-old (20 mo) C57BL/6J mice compared with 3-month-old (3 mo) mice. We evaluated bioenergetic function, redox balance, and calcium buffering, as well as mitochondrial ultrastructure, proteins related to mitochondrial fission and fusion, mitophagy, and biogenesis. Interestingly, mitochondria from the hippocampus of the aged mice presented severe alterations in electron density, with reduced bioenergetics function of the ETC. This is accompanied by increased mitochondrial ROS production, oxidative damage, and inefficient antioxidant response. In addition, aged hippocampal mitochondria have increased levels of calcium and higher sensitivity to calcium overload, possibly due to increased mPTP opening. Finally, the female-aged mice had alterations in mitochondrial dynamics, with an apparent reduction in fission and fusion, mitophagy, and biogenesis processes. Altogether, these explain the accumulation of damaged mitochondria in the hippocampus during aging, which contributes to age-related memory loss and the development of neurodegenerative diseases. 

## 2. Results

### 2.1. Mitochondrial Bioenergetic Dysfunction Is Observed in the Hippocampus of Aged Female C57BL/6J Mice

**Figure 1:**
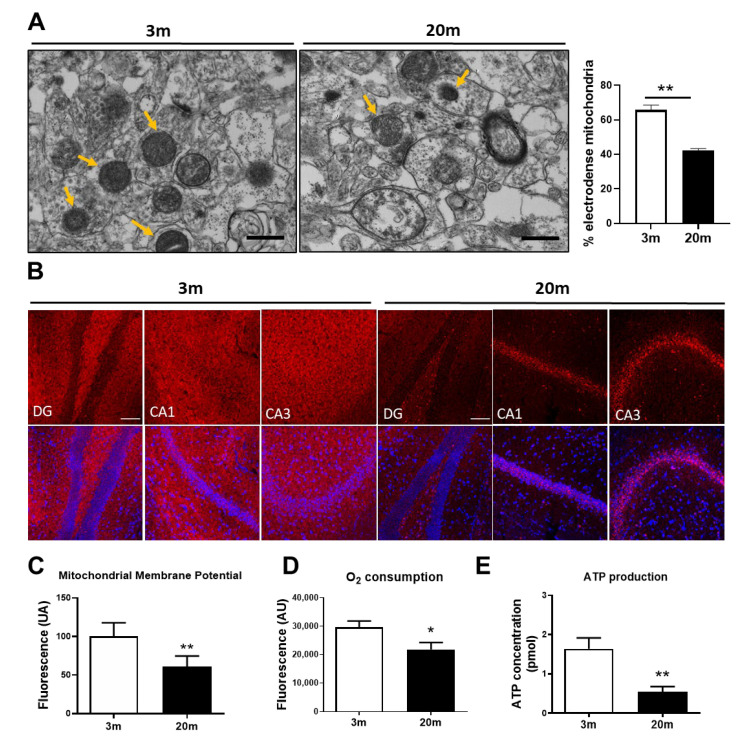
**Hippocampal aging reduces energy production and metabolic power of mitochondria in female C57BL/6J mice.** (**A**) Representative transmission electron microscopy (TEM) images of the hippocampal CA1 region of 3 mo and 20 mo mice with its quantification indicating the percentage of electrodense mitochondria (26,500×). Scale bar corresponds to 500 nm. Yellow arrows show electrodense mitochondria. (**B**) Representative images of non-fixed hippocampal tissue stained with MitoTracker Red CM-H2Xros (MitoRedΨ), as a mitochondrial membrane potential (MMP) indicator. Immunofluorescence images (20×) of three hippocampal regions. DG Dentate Gyrus, CA1 Cornu ammonis 1, CA3 Cornu Ammonis 3. The scale bar corresponds to 100 µm. (**C**) Mitochondrial membrane potential measured by quantitative analysis of fluorescence intensity of MitoRedΨ in an isolated mitochondrial fraction. (**D**) Oxygen Consumption in isolated mitochondria of hippocampal tissue, evaluated with a detection kit. (**E**) ATP content in the hippocampal tissue of 3 mo and 20 mo mice, measured with an ATP Bioluminescence detection kit. *n* = 5 different animals of each age. Graph bars represent means ± SEM. * *p* < 0.005; ** *p* < 0.01.

The main function of mitochondria is to produce energy in the form of ATP, which is fundamental to the proper functioning of several processes within the cells. In neurons, this is especially important for the synaptic process for vesicle recycling, neurotransmitter release, and ATP-dependent ion pumping [5]. Since the ATP production process is highly dependent on the function of the ETC complexes, which are embedded in the cristae of IMM, we evaluated the bioenergetic state of hippocampal mitochondria of 20 mo female C57BL/6J mice compared with 3 mo female C57BL/6J mice, firstly with the evaluation of the percentage of electrodense mitochondria through a quantitative analysis of transmission electron microscopy (TEM) images. We observed a decrease in electrodense mitochondria in the aged mice when compared to the adult mice (Figure 1A), which is in line with the decrease in the expression of complexes I and IV, as we have previously shown [35]. In addition, we measured the MMP in isolated mitochondria and non-fixed hippocampal slices using the fluorescent dye Mitotracker Red CM-H2Xro. In both cases, we observed a decrease in the MMP in the hippocampus of the aged female mice (Figure 1B,C), suggesting that during aging, there is an increase in depolarized mitochondria. We also evaluated oxygen consumption and ATP production of isolated mitochondria, and we observed a decrease in both parameters (Figure 1D,E). Thus, together, these results indicate an impairment in mitochondrial respiration in the hippocampus of female mice during aging.

### 2.2. Redox Imbalance Is Observed in the Aged Hippocampus of Female C57BL/6J Mice

**Figure 2:**
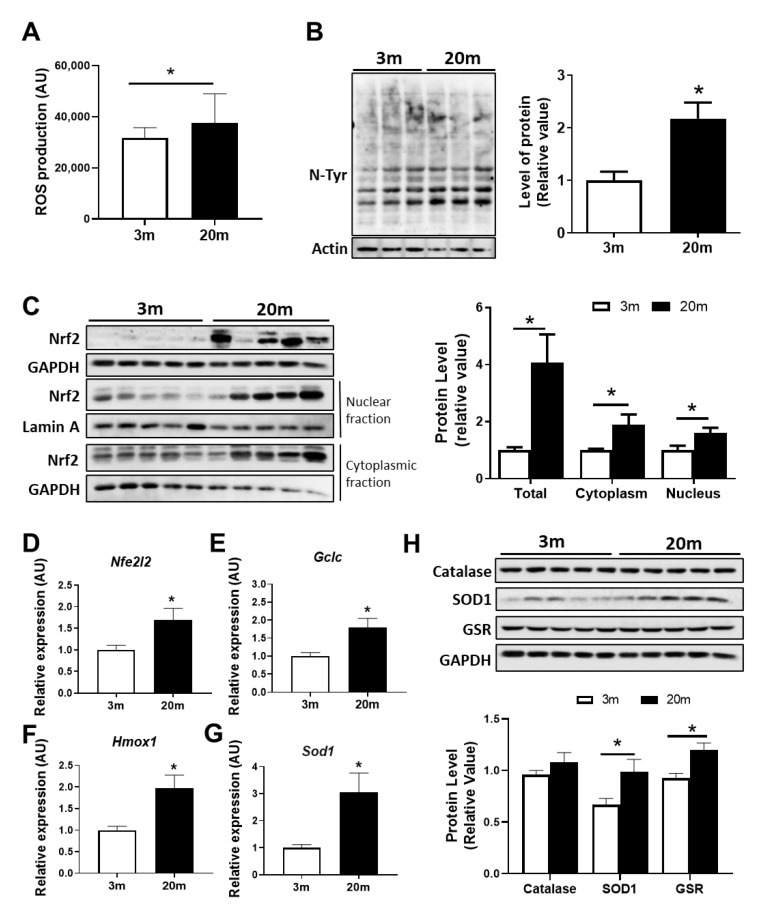
**Oxidative stress and antioxidant responses in the aged hippocampus of female C57BL/6J mice.** (**A**) ROS production of an isolated mitochondrial-enriched fraction measured by CM-H2DCFDA. (**B**) Western blot for nitrotyrosine (N-Tyr) in hippocampal lysate with their densitometric analysis expressed relative to the control. (**C**) Western blot of nuclear factor erythroid 2-related factor 2 (Nrf2) in total lysate, cytoplasmic, and a nuclear fraction of the hippocampal tissue of 3 mo and 20 mo mice with their densitometric analysis. (**D**) Relative mRNA expression of *Nfe2l2* and (**E**–**G**) its downstream target genes, *Gclc*, *Hmox1*, and *Sod1*. (**H**) Western blot for oxidative stress defense in hippocampal with their densitometric analysis. The proteins analyzed were Catalase, Superoxide dismutase 1 (SOD1), and Glutathione Reductase (GSR) * *p* < 0.005, data are presented as the means ± SEM. *n* = 5 different animals per group.

Another important function of mitochondria is redox balance. Mitochondria are the main producer of ROS within the cells since they produce hydrogen peroxide (H_2_O_2_) and superoxide (O_2_^−^) [36]. Superoxide free radicals are produced as a result of the partial reduction in oxygen due to electron leakage from the ETC, and then they are converted to H_2_O_2_ by the mitochondrial superoxide dismutases (SODs) [36]. We evaluated the production of ROS from a hippocampal-enriched-mitochondrial fraction through the fluorescent dye CM-H2DCFDA and observed that the mitochondria of the aged mice produced significantly more ROS than mitochondria from 3 mo mice (Figure 2A). We then evaluated if this increase in mitochondrial ROS induces oxidative damage in the cells, and in order to do so, we measured the levels of nitration of tyrosine residues in a whole lysate from the hippocampus using the antibody anti-nitrotyrosine. We observed an increase in protein nitrotyrosination in the hippocampus of the aged mice compared with the adult mice (Figure 2B), suggesting that the increase in mitochondrial ROS induces protein oxidation in the hippocampus during aging. One key element of the antioxidant response activated by oxidative stress is the Nuclear factor erythroid 2-related factor 2 (Nrf2), a transcription factor that, when activated, translocates to the nucleus and induces the transcription of a variety of genes associated with antioxidant response [37]. We evaluated the levels of this transcription factor in a hippocampal lysate, and we observed increases in Nrf2 protein and mRNA levels in the aged mice (Figure 2C,D). Moreover, we observed an increase in Nrf2 levels in a nuclear and a cytoplasmic fraction (Figure 2C), suggesting the activation of this antioxidant response is due to oxidative stress. We then measured the mRNA levels of different antioxidant enzymes, such as the catalytic subunit of glutamate-cysteine ligase (GCLC), which is involved in the first step of the synthesis of the antioxidant protein glutathione (GSH) [38]; Heme Oxygenase-1 (HO-1), involved in heme-degradation-producing metabolites with antioxidant activity [39]; and Superoxide Dismutase 1 (SOD1), which turns O_2_^−^ into H_2_O_2_. We observed an increment in the mRNA levels of *gclc*, *hmox1*, and *sod1* (Figure 2E–G) in the hippocampus of the 20 mo mice. This is accompanied by an increase in the protein levels of SOD1 and glutathione reductase (GSR) (Figure 2H), which reduce glutathione disulfide to glutathione, suggesting that, during aging, there is an activation of different antioxidant responses. All these results indicate that, during aging, there is increased production of ROS by mitochondria, which leads to oxidative stress and the consequent activation of antioxidant responses.

### 2.3. The Hippocampus of Aged Female Mice Shows Decreased Capacity for Mitochondrial Calcium Regulation

**Figure 3:**
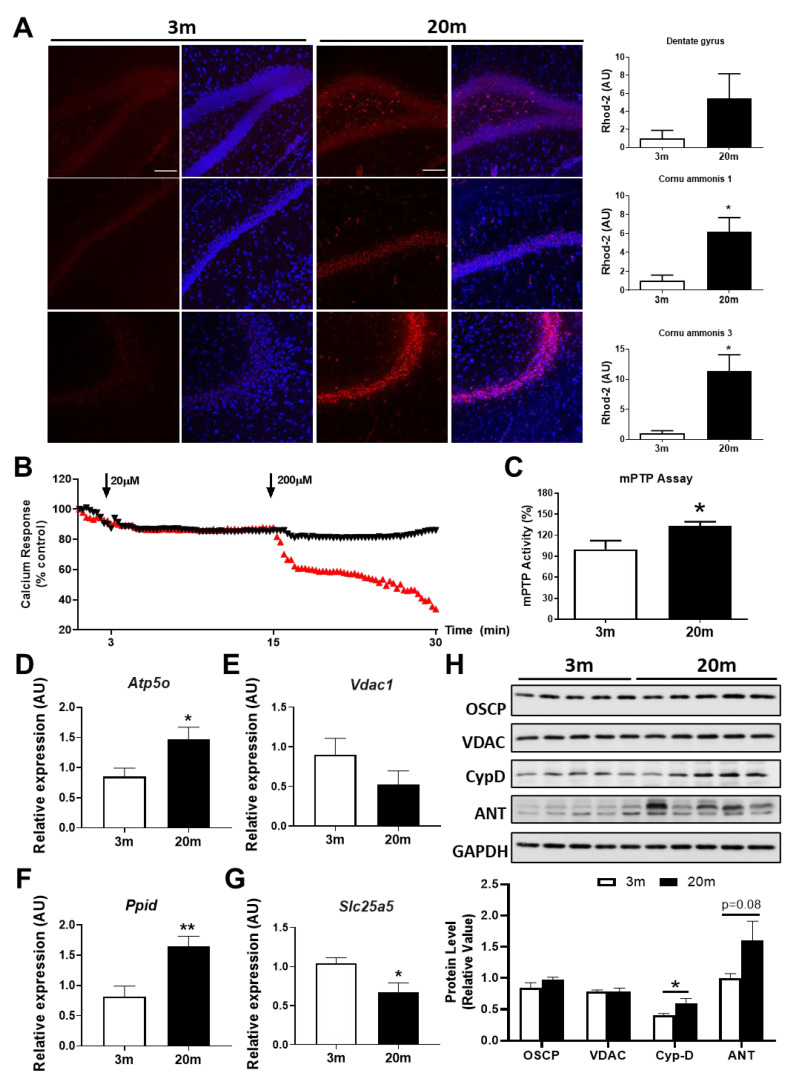
**Hippocampal aging of female C57BL/6J mice is associated with increased mitochondrial calcium levels and reduced calcium buffering.** (**A**) Representative images of non-fixed hippocampal slices stained with the mitochondrial calcium fluorescent dye Rhod-2, as a mitochondrial calcium indicator. Quantitative analysis of fluorescence intensity was performed in the DG, CA1, and CA3 regions of the hippocampus (20×). The scale bar corresponds to 100 µm. (**B**) Measurement of the calcium response by isolated mitochondria after exposure to 20 µM and 200 µM, CaCl_2_. Black triangle: 3 mo mice; Red triangle: 20 mo mice. (**C**) Measurement of mPTP activity in isolated mitochondria through a fluorescent mPTP Assay. (**D**) Relative mRNA expression of *Atp5o*, (**E**) *Vdac1*, (**F**) *Ppid*, and (**G**) *Slc25a5*. (**H**) Western Blot of hippocampal lysates and densitometric analysis of the protein components of the mPTP: OSCP, VDAC, CypD, and ANT. Densitometric analysis is expressed as levels relative to control. Graph bars represent means ± SEM. * *p* < 0.05, ** *p* < 0.01.

Calcium is an important second messenger that is essential for synaptic communication since it is involved in the control of membrane potential and action potential in neurons [40,41]. Specifically, mitochondrial calcium is involved in the regulation of a variety of processes within the cells, such as cell death, cellular signaling pathways, ETC functioning, and mitochondrial ROS production [42,43,44], and calcium deregulation is associated with several neurodegenerative diseases such as AD [45,46,47]. To evaluate the mitochondrial calcium buffering capacity in the hippocampus, we measured the mitochondrial calcium levels via the fluorescent dye Rhod-2 in hippocampal slices of 3 mo and 20 mo mice. We observed that there was an increase in Rhod-2 fluorescence in the dentate gyrus (DG), cornu ammonis 1 (CA1), and cornu ammonis 3 (CA3) hippocampal regions (Figure 3A), suggesting an increase in mitochondrial calcium load and, thus, a decrease in their buffering capacity. In addition, when we measured the calcium response of hippocampal mitochondria, we observed an increase in the susceptibility to swelling of aged mitochondria when they are challenged with 200 µM of calcium (Figure 3B). The increases in swelling and calcium overload are highly associated with the permanent opening of mPTP; therefore, we measured the activity of the mPTP in the hippocampus of the aged mice and there was an increase in the activity of this mitochondrial pore when compared to 3 mo mice (Figure 3C). We also evaluated the mRNA levels of the mPTP components, and we observed an increase in Cyp-D and OSCP and a decrease in ANT mRNA levels (Figure 3D–G). Moreover, when we analyzed the protein levels of these mPTP components, we observed a significant increase in Cyp-D with a clear tendency to increase in ANT (Figure 3H). As Cyp-D has been described as the main regulator of the mPTP opening [48,49,50], the increment in the expression of this protein together with the increase in mitochondrial calcium load may be responsible for increased mPTP activity, contributing to mitochondrial dysfunction in aging.

### 2.4. Altered Levels of Proteins Related to Fission and Fusion Processes Suggest an Imbalance of Mitochondrial Dynamics in the Hippocampus of Aged Mice

**Figure 4:**
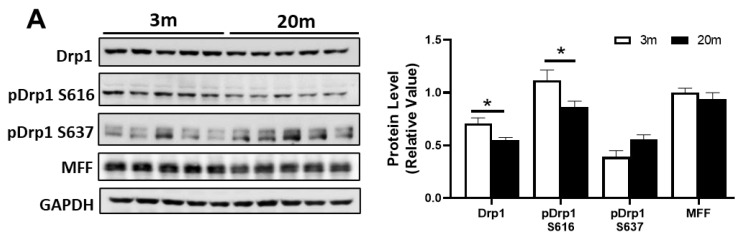

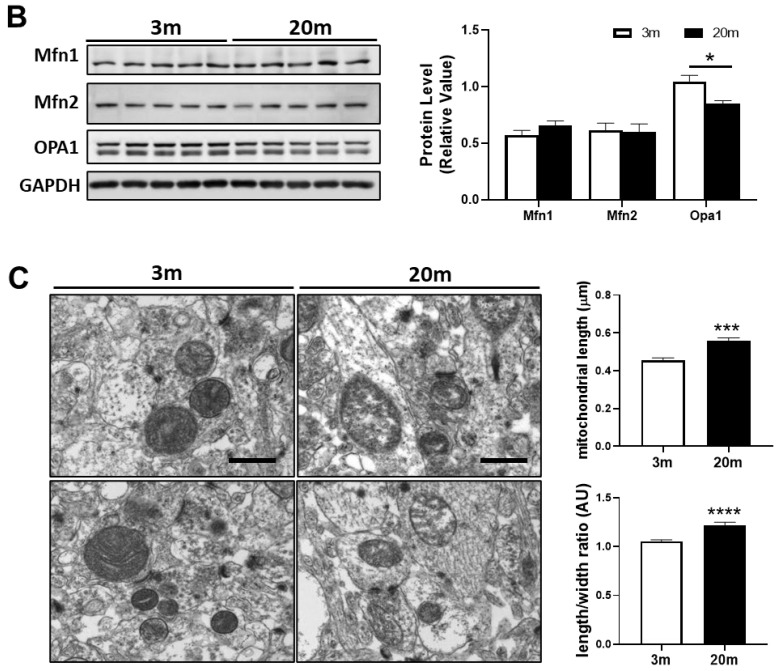
**Changes in proteins implicated in mitochondrial fission and fusion processes in the hippocampus of aged female C57BL/6J mice.** (**A**) Western blot of hippocampal lysates of 3 mo and 20 mo old mice, with their densitometric analysis of proteins involved in mitochondrial fission, including Drp1, pDrp1 S616, pDrp1 S637, and mitochondrial fission factor (MFF). (**B**) Western Blot of the protein involved in mitochondrial fusion, including Mfn1, Mfn2, and OPA1. (**C**) Representative TEM images and analysis showing mitochondrial length and mitochondrial AR in both 3 mo and 20 mo mice (26,500×). The scale bar corresponds to 500 nm. Densitometric analysis is expressed as levels relative to control. Graph bars represent means ± SEM. * *p* < 0.05, *** *p* < 0.005, **** *p* < 0.001.

The balance between fusion and fission is essential for maintaining mitochondrial morphology and function. It has been reported that during age-related neurodegenerative diseases, such as AD, there is an increase in fragmented mitochondria as a result of an increase in the fission process [51,52]. However, the deregulation of mitochondrial dynamics in aging has still not been fully described. Thus, we evaluated the protein expression of fission and fusion proteins in the hippocampus of the 3 mo and 20 mo mice (Figure 4A,B). Surprisingly, we observed, in the aged mice, a significant decrease in Drp1 total levels and also in the phosphorylation of Ser616 of Drp1, a regulatory phosphorylation that enhances Drp1 function. Likewise, we observed a tendency towards an increase in the phosphorylation of Ser637, which inhibits Drp1 activity (Figure 4A). However, we did not observe changes in the protein levels of mitochondrial fission factor (MFF), suggesting that during hippocampal aging, there is a reduction in fission processes due to a decrease in Drp1 but not in the mechanism of recruitment to the mitochondria. Regarding fusion proteins, we observed no changes in Mfn1 and Mfn2, but a decrease in Opa1 levels (Figure 4B) in the 20 mo mice. These results suggest an imbalance in both processes, and as Opa1 is also involved in cristae remodeling, the decrease in this protein could also contribute to the alterations in cristae density and mitochondrial morphology, as shown in Figure 1. In addition, we measured the mitochondrial aspect ratio (AR) in the adult and aged hippocampus, and we found that the mitochondria of the 20 mo mice had a significant increase in the AR due to an increase in their length when compared with the 3 mo mice (Figure 4C). These results suggest an imbalance in both processes, with a decrease in fission and fusion, which may result in more damaged mitochondria since they would not be fusioning to improve function but, also, they would not be fissioning, impeding mitophagy.

### 2.5. Reduced Mitochondrial Mass in the Aged Hippocampus, with Decreased Mitochondrial Biogenesis and Mitophagy

**Figure 5:**
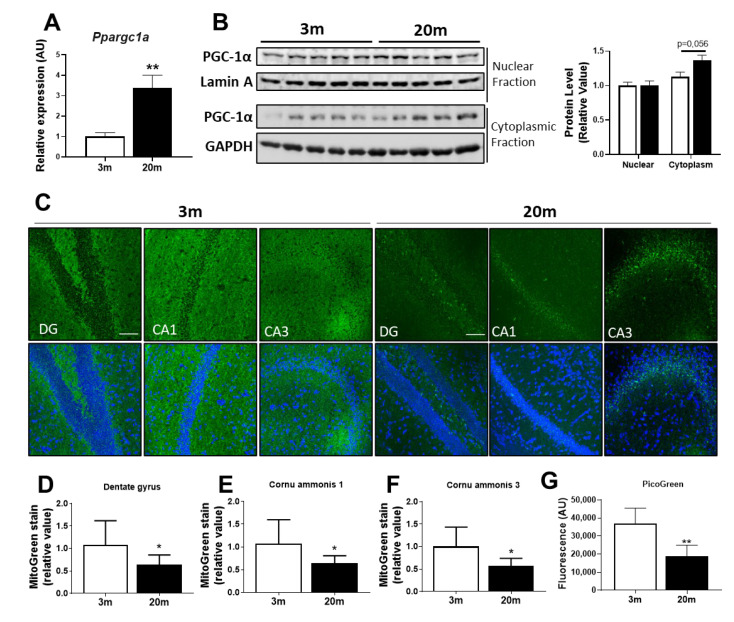
**Mitochondrial biogenesis parameters in the hippocampus of female 3 mo and 20 mo C57BL/6J mice**. (**A**) Relative mRNA expression of peroxisome-proliferator-activated receptor gamma co-activator (*Ppargc1a*) of the hippocampus. (**B**) Western blot and densitometric analysis of PGC-1α levels in a nuclear fraction and a cytoplasmatic fraction. (**C**) Representative images of non-fixed hippocampal slices stained with the mitochondrial fluorescent dye MitoTracker Green FM (MitoGreen) as a measurement of mitochondrial mass. The scale bar corresponds to 100 µm. (**D**–**F**) Quantitative analysis of fluorescence intensity was performed in the DG, CA1, and CA3 regions of the hippocampus (20×). (**G**) PicoGreen dsDNA quantitation assay in an isolated mitochondrial enriched fraction. Densitometric analysis is expressed as levels relative to control. Graph bars represent means ± SEM. * *p* < 0.05; ** *p* < 0.01.

Other important processes that contribute to the proper functioning of mitochondria as a mechanism of quality control are mitochondrial biogenesis, which generates new mitochondria, and mitophagy, which eliminates damaged mitochondria [53]. Several reports show that the deregulation of these processes in the hippocampus occurs in neurodegenerative disease [54,55,56], with controversial results in aging [55]. Regarding mitochondrial biogenesis, the master regulator is PPAR-Gamma-Coactivator-1α (PGC-1α), which regulates other factors, such as Nuclear Respiratory Factors (NRF1 and NRF2) [57]. NRF1 regulates the expression of the mitochondrial transcription factor A (TFAM) [57,58], which is involved in the transcription of mitochondrial-encoded genes. Furthermore, NRF1 and NRF2 are involved in the transcription of the ETC complexes encoded in the nucleus [57]. The coordination between the expression of mitochondrial-encoded and nuclear-encoded ETC complexes is essential for the biogenesis process and proper functioning mitochondrial. We evaluated the mRNA levels of PGC-1α and we observed a significant increase in the aged mice (Figure 5A); however, when we evaluated the hippocampal nuclear and cytoplasmic levels of this protein, we observed an increase in cytoplasmic PGC-1α, with no changes in nuclear levels of PGC-1α (Figure 5B), which may suggest that, during aging, there is an increase in PGC-1α levels, but this protein cannot reach the nucleus to perform its function. Moreover, we evaluated the mitochondrial mass using the mitochondrial dye MitoTracker Green FM in hippocampal slices. We observed a decrease in the fluorescence of MitoTracker Green in the three hippocampal regions DG, CA1, and CA3, where the fluorescence is mainly concentrated in the perinuclear area (Figure 5C–F), suggesting a decrease in the mitochondrial mass. We also evaluated the amount of mtDNA from an isolated mitochondrial fraction, through the Picogreen assay, and we observed a decrease in the mtDNA of the aged mice (Figure 5G), supporting the idea of a decrease in mitochondrial mass.

**Figure 6:**
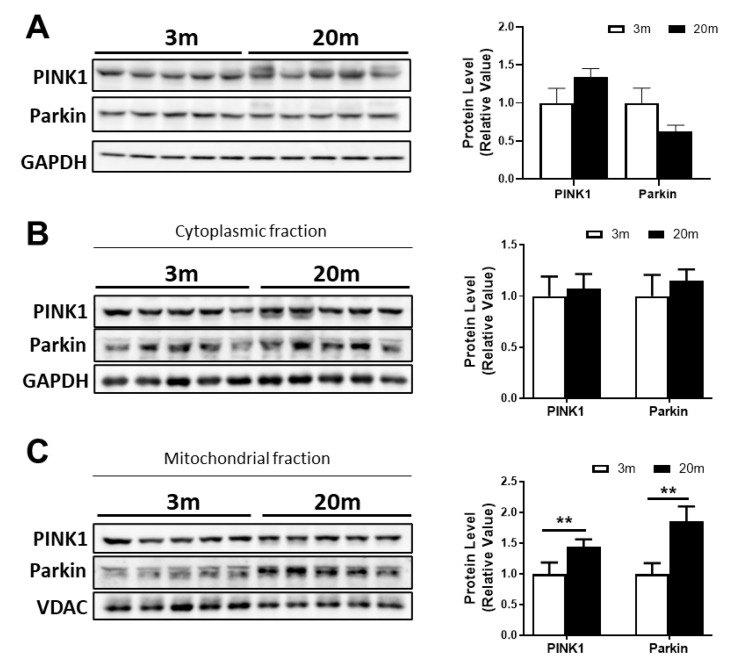
**PINK1 and Parkin levels are increased in the mitochondria of the hippocampus of aged female C57BL/6J mice.** Western blot and densitometric analysis of PTEN-induced kinase 1 (PINK1) and Parkin levels in (**A**) total lysate and (**B**) cytoplasmatic and (**C**) mitochondrial fraction of hippocampus. Densitometric analysis is expressed as levels relative to control. Graph bars represent means ± SEM. ** *p* < 0.01.

Regarding mitophagy, it is a specialized autophagy responsible for the degradation of dysfunctional mitochondria to maintain mitochondrial functionality [59]. There are several mechanisms of mitophagy; however, the classic mechanism involves two main proteins: PINK1 and Parkin [60]. When mitochondria are depolarized, PINK1 is autophosphorylated, leading to the recruitment of Parkin to the outer mitochondrial membrane (OMM), which is also phosphorylated by PINK1. As Parkin is an E3 ubiquitin ligase protein, it induces the ubiquitination of OMM proteins and the recruitment of the autophagic machinery to the damaged mitochondria [60]. We evaluated PINK1 and Parkin levels in a total lysate as a measurement of mitophagy in normal aging, and we did not observe significant changes between the 3 mo and 20 mo mice. Then, we evaluated the mitochondrial and cytoplasmic levels of PINK1 and Parkin and, surprisingly, we observed an increase in both proteins in the mitochondrial fraction (Figure 6B).

Taken together, these data show a complete analysis of mitochondrial dysfunction during normal aging. We have described bioenergetic failure, redox imbalance, deregulation of calcium homeostasis, an imbalance in dynamic processes and impairment in quality control processes, such as mitochondrial biogenesis and mitophagy, which could finally be a contributing factor to the aging phenotype and the development of age-related neurodegenerative diseases.

## 3. Discussion

**Figure 7:**
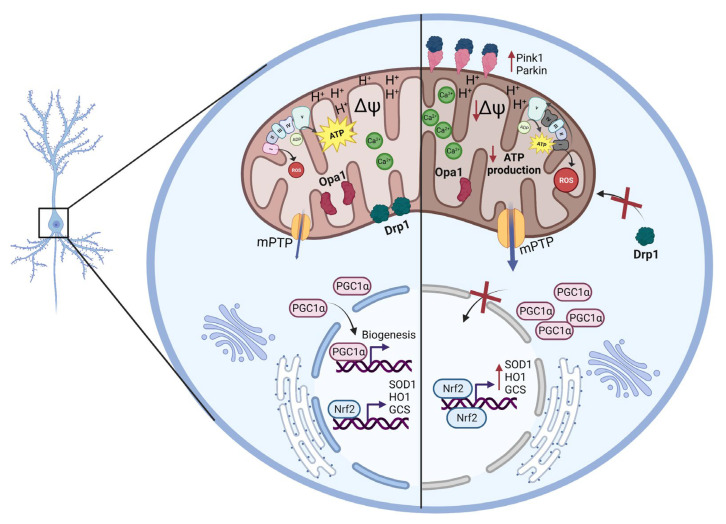
**Summary scheme.** Representative scheme of our main results. We observed that in the hippocampus of female aged mice, there is a reduction in MMP, along with a reduction in ATP production. We also observed an increase in ROS production, antioxidant enzymes, and in calcium concentration within mitochondria. Moreover, we observed a decrease in dynamic proteins, such as Drp1 and its phosphorylation S616, and Opa1. Finally, we observed a decrease in nuclear levels of PGC1α and an increased recruitment of PINK1 and Parkin to the mitochondria.

Understanding aging events at a cellular level is of the utmost importance to tackle healthspan during the later stages of life. Here, we studied mitochondrial-related impairments in female C57BL/6J aged mice to gain a deeper understanding of the mechanisms that may underlie aging processes in the hippocampus. We reported that hippocampal mitochondria were dysfunctional in the aged mice (20 mo) in comparison to the adult mice (3 mo), indicated by a decrease in ATP production and MMP, an increase in calcium sensitivity, an increase in ROS production and protein oxidative damage, and also an imbalance in mitochondrial fusion and fission proteins, as well as altered biogenesis and mitophagy, showing, for the first time in the same study, a complete analysis of the age-related mitochondrial changes in the hippocampus (Figure 7).

We previously reported that hippocampal mitochondria from aged C57BL/6J mice (using females and males and without differentiating between the sexes) show impaired bioenergetic function, due, in part, to a decrease in the expression of complexes I and IV and decreased MMP, evidencing depolarization of hippocampal mitochondria in aged animals [35,61,62]. Similar results have been obtained in studies about aged rats, with decreased activity in complex 1 [62]. A study performed on male 12 mo C57BL/6 mice showed that expression levels of the mitochondrial complex proteins in hippocampal tissue display significant decreases compared to the mitochondria from male 1-month-old C57BL/6 mice [63]. In another study, Wistar rats provided evidence of a significant decrease in mitochondrial bioenergetics with aging [64]. Additionally, complexes I and IV were observed to have reduced enzymatic activity during aging. These characteristics accompanied by MMP reduction have also been observed in aged male animal models, in both rats and mice [65]. Similarly, the analysis of the brain mitochondrial function of male mice of the CD-1/UCadiz strain revealed a decrease in mitochondrial oxygen consumption during aging [66]. Here, we showed that mitochondrial bioenergetic is also severely compromised in female aged C57BL/6J mice where MMP, oxygen consumption, mitochondrial electrodensity, and ATP concentration are reduced. As mitochondrial ATP production depends on the correct assembly and function of ETC complexes and these are located in the IMM, mitochondrial electron density could also be a predictor of mitochondrial respiration or ATP production. Increased density of cristae shows a higher electron density and may allow for a major amount of ETC complexes and, consequently, higher ATP production via aerobic respiration [67,68]. Thus, the reduction in electrodensity of mitochondria that our results shows could be related to a reduction in cristae number, which implies less ETC density, and also a reduction in the formation of supercomplexes in the IMM, which is correlated with mitochondrial bioenergetic dysfunction [69,70,71]. During aging, there is a reduction in both cristae density and supercomplex formation in liver mitochondria; however, there are no changes to heart mitochondria [70], suggesting that these alterations are organ-specific. The reduction in mitochondrial electrodensity in the hippocampus of female C57BL/6J aged mice described in this study suggests a decrease in ETC complexes, which coincides with the reduction in ATP production and bioenergetic status.

The decrease in bioenergetics is correlated with redox imbalance since the production of ROS is mainly due to complex I and III [72]; thus, dysfunctional ETC leads to impairment in ROS production. Several studies have shown that, during aging, there is an increase in ROS levels due to lower levels of mitochondrial antioxidant enzyme activity, such as superoxide dismutase (SOD), and an increase in the expression of NOX2, an enzyme responsible for the generation of superoxide anion [64,73,74], which led to the free-radical theory of aging [75]. In our study, we observed increased ROS production by OXPHOS in the hippocampal mitochondria of the female C57BL/6J aged mice, accompanied by increased nitrotyrosine proteins, supporting the fact that oxidative stress occurs within the aged hippocampus. These results show that during female aging, mitochondrial oxidative stress promotes protein oxidation, in agreement with previous results indicating oxidative damage of not only proteins [76] but also lipids [77], and DNA oxidation [78] contributing to mitochondrial and whole-cell dysfunction in aging [79]. ROS imbalance is related to a reduction in antioxidant proteins [80], while other studies have reported that no alteration in antioxidant protein levels is observed during aging, but antioxidant activity is increased in male aged mice [81]. Here, we report increased levels of Superoxide dismutase 1 (SOD1), and Glutathione Reductase (GSR) protein levels; however, assays related to enzymatic activity are necessary to demonstrate if this increment results in increased antioxidant response. Interestingly, studies carried out on other aged animal models have proven that antioxidant response in aged males is approximately half of that in females [29,82]. These data emphasize that brain aging in male mice and rats is marked by a decrease in antioxidant defense and increased oxidative stress [29,82,83,84]. Thus, it was observed that aged female mice produce 50% of the hydrogen peroxide produced by aged male mice and that the aged females express more antioxidant enzymes in comparison to the males [82]. In the presence of rotenone (a strong inhibitor of complex I of the mitochondrial respiratory chain), aged male rats had higher levels of H_2_O_2_ production compared to female rats [85].

An important antioxidant pathway is governed by the Nrf2 transcription factor. Nrf2 translocates to the nucleus under oxidative stress, promoting antioxidant gene expression, such as GCS, HO1, and SOD1 [86,87]. There is a general consensus that Nrf2 signaling is reduced during aging [88,89]; however, several reports show controversial results of unchanged or increased Nrf2 in the hippocampus of aged male animals [90]. A study on the cerebellum of 21m male mice showed increased transcript and protein levels of Nrf2 with an increase in its antioxidant target genes [91]. Similarly, our data demonstrate that in the hippocampus of female aged C57BL/6J mice, the Nrf2 pathway seems to be activated due to increased protein levels of Nrf2 in the nuclear fraction and increased expression of its target genes. Nevertheless, this Nrf2 activation seems unable to counteract the oxidation, due to the increase in nitrotyrosinated proteins, suggesting that the increase in mRNA and protein levels of the antioxidant enzymes may not be correlated with its activity. Thus, the activation of the Nrf2 pathway could be considered a significant adaptive response to endogenous and exogenous factors in the whole brain in aging. More studies are necessary to validate this possibility.

Another important function of mitochondria is to regulate intracellular calcium concentration via the transient opening of the mitochondrial permeability transition pore (mPTP), mitochondrial calcium uniporter (MCU), and Na^+^/Ca^2+^ exchanger (NCLX) [92,93]. In aging, it is not known which specific calcium homeostasis mechanisms are altered [94], but calcium management is, indeed, impaired [95]. We observed an increase in mitochondrial calcium concentration in the hippocampus of the female aged C57BL/6J mice, accompanied with high calcium sensitivity, evidencing calcium management impairment. Consistent with these findings, past studies from our laboratory showed reduced mitochondrial calcium buffering in the hippocampus of aged C57/B6 mice [61,96]. Further, studies using aged male rats show reduced calcium buffering capacity in response to increased calcium concentration compared to young rats [31]. Therefore, this seems to be a characteristic of hippocampal mitochondria in aged animals. Upon calcium overload in the mitochondrial matrix, sustained mPTP opening is promoted, which induces mitochondrial swelling, dissipation of the MMP, and ultimately cell death [97]. We propose that the calcium overload observed in the aged hippocampal mitochondria could be likely due to an increase in mPTP activity, which is denoted by the mPTP assay and also by an increase in Cyclophilin D (Cyp-D), a master regulator of mPTP opening that is also enhanced after interaction with OSCP [98]. Cyp-D deletion in mice induces less mPTP opening and a better buffering capacity, which makes mitochondria resistant to swelling [48]. Furthermore, we observed a decrease in mRNA levels of ANT but a clear tendency to elevation of protein levels. The increase in protein levels is in line with increased mPTP activity, which is detrimental to cells. Therefore, the decrease in the mRNA could be a response to this mPTP permanent opening. We also observed a decrease in biogenesis processes, which means less production of mitochondrial content, including mitochondrial proteins, as we also observed a slight decrease.

Mitochondria form an interconnected network in the cells and are regulated by fusion and fission processes in response to diverse conditions in the cells, such as changes to nutrient availability, signaling, and different stress conditions [99]. Many researchers have proposed that mitochondrial fission and fusion functions are altered in aged brains but, until now, only a few studies have focused on mitochondrial dynamics [26,99]. For example, Drp1 has been shown to decrease in the aged brain in a global proteomic study, but Mfn1 was elevated in the same aged group, consistent with a pro-fusion state [100]. Another study of a mouse model of accelerated senescence SAMP8 shows that Drp1, Mfn2, and OPA1 all decreased in the hippocampus of these mice, also showing a decrease in the processes of fusion and decreased mitochondrial fission [101]. These results are consistent with what we observed in the female aged C57BL76J mice, where Drp1 and Opa1 were reduced in hippocampal samples. Drp1 needed different proteins to be recruited to the mitochondrial membrane, such as MFF, Mid49, and Mid51, among others [102,103]. As we did not observe changes in MFF, we suggest that the reduction in fission processes could be due to the expression and regulation of Drp1 more than impairment in the machine recruitment; however, analysis of the other proteins is still needed. The reduction in Opa1 is in line with the decrease that we observed in the electrodensity of aged mitochondria since Opa1 plays a key role in the formation and dynamics of mitochondrial cristae [104]; therefore, a decrease in this protein could lead to a reduction in the definition of mitochondrial cristae, and, thus, a reduction in mitochondrial electrodensity. In addition, we also observed that female aged mitochondria have an increase in aspect ratio (AR), suggesting that these mitochondria are longer and more elliptical. Since our data show a decrease in Drp1 and Opa1 levels but not in Mfn1 and Mfn2, these more elongated mitochondria could be due to a decrease in the fission process [105]. Moreover, as we have demonstrated previously and again show now, these elongated mitochondria are more damaged, with an increase in swelling [35], suggesting that the increase in the AR may be more related to an increase in damage alongside a decrease in fission than an increase in beneficial fusion processes. In contrast, in a pilot study of aging, Fischer 344 Brown Norway rats showed an increase in Drp1 and Mfn2 protein levels in the liver [26,106]; therefore, the changes in proteins related to fission and fusion processes may be dependent of the tissue analyzed.

PGC1α is a transcriptional coactivator that binds to transcription factors such as Nrf2 to promote mitochondrial biogenesis [107]. Our results demonstrate that cytoplasmic levels of PGC1α transcript and protein in aged animals are increased compared to adult mice, but there was no change in protein levels in the nucleus, where Nrf2 promotes increased expression of mitochondrial biogenesis genes [108]. Quantitative proteomics studies in aged mice predicted that PGC1a should be activated [100], which is in line with the increase in cytoplasmic levels of this protein. However, studies have shown that PGC1α translocation into the nucleus is regulated. A study in adult male mouse hippocampus shows that increased protein levels of sirtuin 1, a deacetylase that promotes deacetylation and activation of PGC1α, increase the activation of transcriptional co-activator function of PGC1α [109]. On the other hand, a study on aged fibroblasts shows that decreased protein levels of sirtuin 1 promote a deficit in PGC1α activation, resulting from decreased mitochondrial biogenesis [110]. Thus, the increased level of PGC1α in the cytoplasm will be unable to entirely translocate to the nucleus, generating reduced mitochondrial biogenesis in aged animals. Mitotracker Green dye was used to alternatively test mitochondrial biogenesis by mitochondrial mass measurement. This dye selectively accumulates in the mitochondrial matrix where it covalently binds to mitochondrial proteins. Here, we show that not only is mitochondrial mass reduced in the hippocampus of aged animals, but also mtDNA content is diminished, as measured by Picogreen assay in a mitochondrial fraction. These observations were found in all the regions of the hippocampus, as we and others have suggested before [35,111,112], supporting that mitochondrial biogenesis seems to be hindered in our aged animals. Interestingly, and in concordance with our results, a study on aged male Wistar rats determined that mitochondrial mass significantly decreases by 19% in the hippocampuses of the aging male rats, and this decrease was counteracted with high doses of vitamin E, which improved mitochondrial biogenesis and increased hippocampal mitochondrial mass [29].

The clearance of damaged mitochondria by mitophagy is of the utmost importance to maintain cellular homeostasis [113]. PINK1 and Parkin-mediated mitophagy are some of the most studied mechanisms [114]. This mitophagy pathway relies on Parkin being phosphorylated by PINK1, thus promoting Parkin recruitment to the mitochondria to trigger mitophagy [115]. Hence, mitochondrial localization of these proteins is important for this process. We studied PINK1 and Parkin expression and mitochondrial localization and found that both proteins are increased in the mitochondria, suggesting that mitophagy increased in our female aged mice. Other reports show that mitophagy is reduced by up to 70% in the hippocampus of aged mice [116]. This was measured in a transgenic mice model expressing mt-Keima, a protein that exhibits pH-dependent excitation and lysosomal resistance, which serves as a mitophagy fluorescent reporter. Therefore, these data suggest that the localization of mitochondria within the lysosome is decreased, which, alongside our data, could indicate that there is an induction of mitophagy in aging, leading to the accumulation of PINK1 and Parkin in the mitochondria. However, mitophagy is not complete due to an impairment in the autophagic flux. Thus, we concluded that, during aging, both processes are decreased, which could be contributing to the accumulation of damaged mitochondria, and that their modulation may contribute to the mitochondrial improvement necessary to maintain good mitochondrial and cellular functioning. Nevertheless, further studies are necessary to better identify the mechanisms of interaction between mitochondrial functions and aging and analyze possible factors influencing these processes.

## 4. Materials and Methods

Animals. Female 3-month-old mice and 20-month-old mice C57BL/6J were obtained from Fundación Ciencia and Vida and the Institute of Public Health (ISP) in Chile. These animals were housed and maintained at 24 °C on a 12:12 h light–dark cycle, with food and water provided ad libitum. The animals were handled according to the National Institute of Health guidelines (NIH, Baltimore, MD, USA). The experimental procedures were approved by the Bioethical and Biosafety Committee of Universidad San Sebastián. The animals were anesthetized using isoflurane in an anesthesia chamber and then euthanized by decapitation. The experimental procedures were approved by the Bioethical and Biosafety Committee of Universidad San Sebastián. This study was carried out in compliance with the ARRIVE guidelines.

Reagents and antibodies. The primary antibodies used were mouse anti-β-actin (1:1000, sc-47778, Santa Cruz Biotechnology, Inc., Dallas, TX, USA), rabbit anti-GAPDH (1:1000, sc-25778, Santa Cruz Biotechnology, Inc., USA), mouse anti-Nitrotirosine (1:1000, sc-32757, Santa Cruz Biotechnology, Inc., USA), rabbit anti-Nrf2 (1:1000, sc-722, Santa Cruz Biotechnology, Inc., USA), mouse anti-catalase (1:1000, sc-271803, Santa Cruz Biotechnology, Inc., USA), mouse anti-SOD1 (1:1000, sc-271014, Santa Cruz Biotechnology, Inc., USA), mouse anti-Glutathione reductase (1:1000, sc-133245, Santa Cruz Biotechnology, Inc., USA), mouse anti-VDAC (1:1000, sc-390996, Santa Cruz Biotechnology, Inc., USA), mouse anti-OSCP (1:1000, sc-365162, Santa Cruz Biotechnology, Inc., USA), mouse anti-CYP-D (1:1000, sc-37606, Santa Cruz Biotechnology, Inc., USA), mouse anti-ANT (1:1000, sc-293434, Santa Cruz Biotechnology, Inc., USA), rabbit anti-OPA1 (1:1000, mAb 80471, Cell Signaling Technologies, Danvers, MA, USA), mouse anti-MFN1 (1:1000. sc-166644, Santa Cruz Biotechnology, Inc., USA), rabbit anti-MFN2 (1:1000, mAb 11925, Cell Signaling Technologies), rabbit anti-pDRP1 (1:1000 mAb 6319; mb 4494, Cell Signaling Technologies, USA), mouse anti-DRP1 (1:1000, sc-271583, Santa Cruz Biotechnology, Inc., USA), rabbit anti-MFF (1:1000, mAb 84580, Cell Signaling Technologies, USA), mouse anti-biogenesis cocktail (1:1000, ab 123545, Abcam, Cambridge, UK), mouse anti-PGC-1α (1:1000, sc-517380, Santa Cruz Biotechnology, Inc., USA), mouse anti-PINK1 (1:1000, sc-517353, Santa Cruz Biotechnology, Inc., USA), and mouse anti-PARKIN (1:1000, sc-32282, Santa Cruz Biotechnology, Inc., USA). The fluorescent dyes used were: MitoTracker^TM^ Green FM (Catalog number: M7514, Thermo Fisher Scientific, Waltham, MA, USA), MitoTracker Red CM-H2Xros (Catalog number: M7513, Thermo Fisher Scientific, USA), CM-H2DCFDA (catalog number C6827, Thermo Fisher Scientific, USA), Rhod-2 AM, cell-permeant (Catalog number: R1245MP, Thermo Fisher Scientific, USA), and VECTASHIELD Antifade Mounting Medium with DAPI (Catalog number: H1200, Vector Laboratories, Inc., Burlingame, CA, USA).

Immunoblotting. The hippocampus of the 3- and 20-month-old (mo) mice was dissected on ice and immediately processed as previously described [35,96,117]. The hippocampal tissue was briefly homogenized in HEPES buffer (25 mM Hepes, 125 mM NaCl, 25 mM NaF, 1 mM EDTA, 1 mM EGTA, 1% NP-40, pH = 7,4), supplemented with a protease inhibitor mixture (catalog number 78429, Thermo Fisher Scientific) and phosphatase inhibitors (NaF 25 mM, Na_2_P_2_O_7_ 30 µM, Na_3_VO_4_ 100 mM) using a homogenizer and then sequentially passed through syringes of different calibers. The protein samples were centrifuged at 14,000 rpm for 20 min at 4 °C. The protein concentrations were determined using the BCA Protein Assay Kit (Catalog number 23225, Pierce, Rockford, IL, USA). Samples were resolved by SDS-PAGE, followed by immunoblotting on PVDF membranes. The membranes were incubated with the primary antibodies and anti-mouse or anti-rabbit IgG peroxidase-conjugated antibodies (Pierce) and visualized using an ECL kit (Luminata Forte Western HRP substrate, Millipore, Burlington, MA, USA).

Isolation of an enriched-mitochondrial fraction from the hippocampus. As previously described, a fraction enriched in mitochondria was isolated from the hippocampus [35,61,87]. Hippocampal tissue was briefly suspended and lysate in MSH buffer (230 mM mannitol, 70 mM sucrose, 5 mM Hepes, pH 7.4), supplemented with 1 mM EDTA and protease and phosphatase inhibitor cocktail in a glass homogenizer. Homogenates were centrifuged at 600× *g* for 10 min at 4 °C. The supernatant was centrifuged at 8000× *g* for 10 min; the newly enriched-mitochondrial pellet was washed twice in MSH without EDTA. Protein concentration was determined by using a standard BCA kit (Thermo Fisher Scientific, USA).

Transmission Electron Microscopy (TEM). The 3- and 20-month-old mice were perfused with paraformaldehyde 4% and then their brains were removed (*n* = 3) and immediately processed as previously described [35]. The hippocampal coronal sections were obtained and post-fixed with 2.5% glutaraldehyde. Later, the samples were processed and visualized according to the recommendations of the Facility from the Pontificia Universidad Católica de Chile. The hippocampal CA1 region was isolated and fixed in 3% glutaraldehyde in 50 mM cacodylate buffer (pH 7.2) for 3 days at room temperature and then post-fixed with 1% osmium tetroxide in cacodylate buffer for 90 min. The slices were then treated with 1% aqueous uranyl acetate, dehydrated in acetone, and embedded in Epon resin. Ultrathin sections were placed on 300-mesh copper electron microscopy grids, stained with uranyl acetate, and examined in a Phillips Tecnai 12 transmission electron microscope (Philips Electron Optics, Holland) at 80 kV. Morphometric analyses of TEM images were performed with Fiji software. For morphological analysis, we used the Aspect Ratio (AR) parameter, which corresponds to the length of the major and minor axis of mitochondria [118,119].

Hippocampal slice staining with mitochondrial fluorescent dyes. The brains of the 3- and 20-month-old mice were dissected and immediately frozen at −150 °C. The frozen brains were mounted using an optimal cutting temperature compound (OCT compound) in a cryostat at −22 °C; then, coronal 25 µm-thick slices of unfixed hippocampal tissue were obtained. Hippocampal slices were mounted on glass slides and incubated as previously described with mitochondrial fluorescent dyes [35,96,120]. First, the slices were washed three times for 5 min in PBS and then incubated with MitoTracker Green FM to measure mitochondrial mass [96,120,121], MitoTracker Red CM-H2Xros to determine mitochondrial membrane potential [96,120,121] or Rhod-2 AM [121] to measure mitochondrial calcium. All these dyes were diluted in Krebs-Ringer-Hepes-bicarbonate (KRH) buffer (136 mM NaCl, 20 mM HEPES, 4.7 mM KCl, 1.5 mM MgSO_4_, 1.25 mM CaCl_2_, 5 mM glucose; pH = 7.4) and incubated for 45 min at 37 °C. After incubation, slices were washed three times for 5 min in PBS and mounted with fluorescent mounting media with DAPI (Vector Laboratories Inc., USA).

Oxygen Consumption Assay. Oxygen consumption was measured in 50 ug of proteins from an enriched-mitochondrial fraction incubated with oxidative substrates for 30 min a 37 °C, using an Extracellular O_2_ Consumption Assay kit (ab197243, Abcam, UK). In this assay, the fluorescence obtained is proportional to O_2_ consumption generated by the mitochondria.

Measurement of ATP content and MMP. ATP concentration was measured in tissue lysates obtained with HEPES buffer (25 mM HEPES, 125 mM NaCl, 25 mM NaF, 1 mM EDTA, 1 mM EGTA, 1% NP-40, pH = 7,4) using a luciferin/luciferase bioluminescence assay kit (ATP determination kit no. A22066, Molecular Probes, Invitrogen, USA) [96]. The amount of ATP in each sample was calculated from standard curves and normalized to the total protein concentration. MMP was measured in a mitochondrial-enriched fraction (50 μg) diluted in 100 μL of KCl respiration buffer and incubated at 37 °C for 30 min with MitoTracker Red CM-H2Xros. Samples were centrifuged and the fluorescence was measured at 590 nm in the resuspended mitochondrial pellet.

Measurement of ROS production. ROS production was measured using the fluorescent dye CM-H2DCFDA (catalog number C6827, Thermo Fisher Scientific, USA). Briefly, a fraction enriched in mitochondria (25 μg of protein) was isolated from the hippocampus and diluted in 100 μL of KCl respiration buffer with pyruvate and malate as oxidative substrates and the addition of 25 μM DCF. Then, it was incubated at 37 °C for 30 min and centrifuged at 8000× *g* for 10 min at 4 °C. After this time, mitochondrial ROS production was measured in BioTek Synergy HT (485 nm, 530 nm) [87].

Measurement of the calcium response. The mitochondrial response to calcium overload was measured by absorbance to 540 nm (30 °C) [122] for 3 min (basal), and then 20 μM CaCl_2_ was added, and the response was evaluated for 15 min. Next, we added 200 μM CaCl_2_ and measured it for 15 min [87].

Mitochondrial permeability transition pore (mPTP) assay: Mitochondrial permeability transition pore (mPTP) openings were measured in a fraction enriched in mitochondria (50 μg of protein), which were loaded with the acetoxymethyl ester of calcein dye, calcein AM, that passively diffuses into the cells and accumulates in cytosolic compartments, including the mitochondria. In addition, CoCl_2_ was added. The fluorescence from cytosolic calcein is quenched by the cobalt; in contrast, in a condition of closed mPTP, mitochondrial fluorescence is maintained. However, when the mPTP is open, loss of green mitochondrial calcein fluorescence occurs [123].

Mitochondrial DNA measurement. The mitochondrial DNA was measured in a fraction enriched in mitochondria (10 μg of mitochondrial protein) incubated with Quant-iTPicoGreen dsDNA reagent. This reagent emits a fluorescent signal upon binding to nucleic acids (P11496, Invitrogen, USA).

Gene expression by quantitative real-time PCR. Total RNA was isolated from the whole hippocampus. Samples were homogenized in Trizol according to the manufacturer’s instructions. Total RNA was precipitated and treated with a turbo DNase I unit (Invitrogen, USA) to eliminate DNA contamination. Five micrograms of total RNA were reverse transcribed using Super-Script IV reverse transcriptase kit (8090010, Invitrogen, USA) with random hexamers and Oligo d(T)18 to measure the relative expression of *Hmox1*, *Gclc*, *Nfe2l2*, *Ppid*, *Slc25a5*, *Atp5o*, *Vdac1*, *Ppargc1a*, *Cycs*, and *Sod1* genes. The cDNA was quantified by qPCR using Brilliant II SYBR Green and Kapa SYBR fast (Kapa Biosystems, Wilmington, MA, USA). The qPCR analysis was performed in duplicate from a reverse-transcribed product using the Rotor-Gene Q (Qiagen, Germany). Expression changes were calculated following the 2-ΔΔCt method using cyclophilin-A (Cyc) as a normalization control. The primers used are listed in Table 1.

Image analysis. All slides were photographed and scanned under the same magnification, laser intensity, brightness, and gain. Images were processed using Fiji software (NIH Image), adjusting the fluorescence threshold intensity in every picture.

Statistical analysis. The results are presented as bar graphs indicating the mean ± standard deviation. Statistical significance was determined using a *t*-test. *p*-values > 0.05 and ≤0.05 were regarded, respectively, as not statistically significant and as statistically significant. In the figures, *p*-values between 0.01 and 0.05 are marked with one asterisk, *p*-values between 0.001 and 0.01 with two asterisks, and *p*-values less than 0.001 are shown with three asterisks. All statistical analyses were performed using Prism software (GraphPad Software, Inc.).

## 5. Conclusions

This study is of great interest since it shows a great variety of alterations in the hippocampal mitochondria of female C57BL/6L mice at a certain age (20 mo) and allows us to conclude that, during aging, mitochondria lose their capacity to produce energy, regulate ROS production and oxidative stress, and to balance calcium levels. This, together with deficiencies in mitochondrial dynamics and quality control processes, triggers the accumulation of damaged mitochondria in the hippocampus, which could contribute significantly to synaptic and cognitive impairment observed at advanced age.

## Figures and Tables

**Table 1:** qPCR primers.

Gene	Forward Primer	Reverse Primer
*Hmox1*	5′-CACAGCACTATGTAAAGCGTCT-3′	5′-TGTGCAATCTTCTTCAGGACC-3′
*Gclc*	5′-GGGGTGACGAGGTGGAGTA-3′	5′-GTTGGGGTTTGTCCTCTCCC-3′
*Nfe2l2*	5′-ACCCGAAGCACGCTGAAGGC-3′	5′-GTCACTGAACCCAGGCGGTGG-3′
*Ppid*	5′-AGGAGATAGCCCCAGGAGAT-3′	5′-TTGCATACACGGCCTTCTCTT-3′
*Slc25a5*	5′-CCACCCAGGCTCTCAACTTT-3′	5′-AAGCACAAGGATGTAGCCCC-3′
*Atp5o*	5′-CAAGCGCACCGTCAAAGTG-3′	5′-GCACCGTCTTTAACTCAGAGAG-3′
*Vdac1*	5′-AGTAACACTCGCTTCGGAATAG-3′	5′-TGGTTTTAGGGTCTGAGTGTAC-3′
*Ppargc1a*	5′-ACAGAGACACTGGACAGTCT-3′	5′-CATTGTAGCTGAGCTGAGTG-3′
*Cycs*	5′-GTTCAGAAGTGTGCCCAGTG-3′	5′-TACTCCATCAGGGTATCCTC-3′
*Sod1*	5′-ACTTCGAGCAGAAGGCAAGC-3′	5′-AACATGCCTCTCTTCATCCG-3′

## Data Availability

For all the data supporting the reported results, please contact the corresponding author of the study.
